# Crystal Structure and Antitumor Activity of the Novel Zwitterionic Complex of tri-*n*-Butyltin(IV) with 2-Thiobarbituric Acid

**DOI:** 10.1155/2008/654137

**Published:** 2008-04-06

**Authors:** Vasilios I. Balas, Sotiris K. Hadjikakou, Nick Hadjiliadis, Nikolaos Kourkoumelis, Mark E. Light, Mike Hursthouse, Apostolos K. Metsios, Spyros Karkabounas

**Affiliations:** ^1^Section of Inorganic and Analytical Chemistry, Department of Chemistry, University of Ioannina, Ioannina 45110, Greece; ^2^Department of Chemistry, University of Southampton, Highfield, Southampton SO17 1BJ, UK; ^3^Department of Experimental Physiology, Medical School, University of Ioannina, Ioannina 45110, Greece

## Abstract

A novel tri-*n*-butyl(IV) derivative of 2-thiobarbituric acid (HTBA) of formula [(*n*-Bu)_3_Sn(TBA) H_2_O] (1) has been synthesized and characterized by elemental analysis and ^119^Sn-NMR and FT-IR spectroscopic techniques. The crystal structure of complex **1** has been determined by single crystal X-ray diffraction analysis at 120(2) K. The geometry around Sn(IV) is trigonal bipyramidal. Three *n*-butyl groups and one oxygen atom from a deprotonated 2-thiobarbituric ligand are bonded to the metal center. The geometry is completed with one oxygen from a water molecule. Compound **1** exhibits potent, in vitro, cytotoxicity against sarcoma cancer cells (mesenchymal tissue) from the Wistar rat, polycyclic aromatic hydrocarbons (PAH, benzo[a]pyrene) carcinogenesis. In addition, the inhibition caused by **1**, in the rate of lipoxygenase (**LOX**) catalyzed oxidation reaction of linoleic acid to hyperoxolinoleic acid, has been also kinetically and theoretically studied. The results are compared to that of cisplatin.

The increasing interest in the bioinorganic chemistry of organotin(IV) compounds has led to extended studies on their interactions with different types of biomolecules such as carbohydrates, nucleic acid derivatives, amino acids and peptides [1, 2]. The organotin(IV) compounds are exhibiting significant antitumor activity [1–8]. More particularly, organotin(IV) complexes with ligands containing phenolic–OH groups and a heterocyclic nitrogen {N} donor atom comprise an interesting class of such complexes because they contain an amide group [9, 10]. Surprisingly, only few organotin(IV) complexes of this type have been structurally characterized up to now [10]. Recently, the inhibition caused by organotin(IV) complexes of thioamide ligands towards lipoxygenase (**LOX**) catalyzed oxidation reaction of linoleic acid to hyperoxolinoleic acid in relation with the antitumor activity caused by these complexes was studied [3–8] and a mechanism of free radicals was proposed. 2-Thiobarbituric acid, on the other hand, is a reagent in use for the detection of lipid hydroperoxides and lipid oxidation [11, 12].

In order to investigate further the mechanism of cytotoxic activity of organotin(IV)-thioamide complexes, we report here the synthesis of a new complex with formula [(*n*-Bu)_3_Sn(TBA)*⋅*H_2_O] (**1**) (HTBA is the 2-thiobarbituric acid) (Scheme 1). The complex has been characterized by elemental analysis and ^119^Sn-NMR and FT-IR spectroscopic techniques. The structure of the complex was also determined by X-ray crystallography at 120(2) K. The tri-*n*-butlyltin derivative was chosen, since it is known to possess antiparasitic properties [1, 2]. 2-Thiobarbituric acid, a well-known reagent in use for the determination of the lipid peroxidation in biological systems, is chosen in order to possibly increase the LOX inhibition activity of organotin moiety [3–8, 11, 12]. The anticancer cell screening results of the compound tested are also reported. The inhibition caused by **1**, in the rate of lipoxygenase **(LOX)** catalyzed oxidation reaction of linoleic acid to hyperoxolinoleic acid has also been kinetically and theoretically studied.

Complex **1** was synthesized by reacting a methanolic solution of tri-*n*-butlyltin(IV) chloride (*n*-Bu)_3_SnCl with an aqueous solution of 2-thiobarbituric acid (HTBA) which contains an equimolar amount of potassium hydroxide. The structure was solved by direct methods SHELXS97 [13] and successive difference Fourier syntheses. Refinement applied full-matrix least-squares methods SHELXL97 [14]. Atomic scattering factors for neutral atoms and real and imaginary dispersion terms were taken from International Tables for X-ray Crystallography [15]. Intensity data for the colorless crystals were collected on a Nonius Kappa CCD diffractometer with graphite-monochromated MoK*α* radiation at 120(2) K. C_16_H_32_N_2_O_3_SSn, MW = 451.19, monoclinic in *P*2_1_/*n*, *a* = 11.0956(2), *b* = 17.3425(4), *c* = 11.1879(2) Å, *β* = 95.3080(10)°, *V* = 2143.60(7) Å^3^, *Z* = 4, *D* = 1.398 Mg/m^3^, *μ* = 1.303 mm^−1^, final *R* = 0.0247 for 4906 unique observed [*F^2^* > 2 *σ* (*F^2^*)] diffractometer data. Measurements of in vitro cells toxicity have been carried out in preliminary repetitions according to the method described in literature [3–8].

The ir spectrum of complex **1** shows distinct absorption at 2959 cm^−1^ due to the C–H bond vibrations of *n*-butyl groups, at 1546 and 1398 cm^−1^, which are assigned to *ν*(CN) vibrations (thioamide I and II bands) and at 1194 and 906 cm^−1^, which are attributed to the *ν*(CS) vibrations (thioamide III and IV bands). The corresponding thioamide I and II bands in crystalline HTBA appear at 1527 and 1350 cm^−1^ while thioamide bands III and IV are observed at 1154 and 801 cm^−1^ [16].

^119^Sn-NMR spectrum of complex **1,** in DMSO-*d_6_* solution, shows a resonance signal at 5.7 ppm indicating a four coordinated Sn atom [17]. Since the corresponding value for the (*n*-Bu)_3_SnCl in DMSO-*d_6_* is found to be at 2.7 ppm [18], this is indicative of a partial positive charged Sn atom in **1** (see crystal structure). Chemical shifts *δ* are given in ppm referenced to^119^Sn-Me_4_Sn. The stability of **1** in DMSO-*d_6_* solution was verified by recording its ^1^H-NMR spectrum, as a function of time. Free HTBA ligand shows a resonance signal at 12.1 ppm (s, H(N)), 4.9 ppm (s, H(C)), and 3.5 ppm (s, H(C)) (Scheme 1) [16] which are shifted at 10.9 ppm (s, H(N)) and at 4.2 ppm (s, H(C)) in case of **1** . The signal at 3.5 ppm (s, H(C)) could not be observed in the ^1^H-NMR of the complex since enol-keton tautomerims can not be established in case of coordinated HTBA. Resonance signals at 1.55 ppm (t), 1.28 ppm (m), 1.09 ppm (m), and at 0.87 ppm (t) were assigned to the H(C) of the *n-*butyl group.

A diagram of **1** as well as selected bond lengths and angles are shown in Figure 1(a).

The structure of compound **1** consists of one (*n*-Bu)_3_Sn(IV) moiety bonded with a de-protonated 2-thiobarbituric acid (HTBA) molecule and a water molecule. The Sn1 atom exhibits a distorted trigonal bipyramidal configuration with C (5), C (9), and C (13) occupying the equatorial and O2 from HTBA ligand and O3 from the water molecule, occupying the axial positions. According to Reedijk's geometric parameter (*τ* = (*β* − *α*)/60 where *α* is the greatest and *β* the second greatest bond angle around the metal center) [19] the calculated *τ* value is 0.94, being equal to zero for perfectly tetragonal pyramidal geometry and unity for perfectly trigonal pyramidal [19].

The Sn1–O2 bond distance of 2.2287(14) Å is in accordance with that found in [Ph_2_(pyO)SnCH_2_Sn(OH)Ph_2_]_2_ (where pyO = anion of 2-hydroxypyridine), [10] (Sn–O = 2.227(2) Å). The two C–O bond distances (C1–O2 = 1.274(2), and C3–O1 = 1.261(2) Å, resp.) are almost equal. The C1–C2 and C2–C3 bond lengths of 1.386(3) and 1.394(3) Å, respectively, are also equal. This bond distribution in **1** (shown in Scheme 2) leads to a zwitterionic form of the compound (Scheme 2) and is in agreement with ^119^Sn-NMR, in DMSO-*d_6_* solutions. The molar conductance (Λm) value of the complex in DMSO solution (5 10^−3^M) is 5.3 (cm^−1^mole^−1^Ω^−1^) showing that the complex is not conducting in solution confirming the stability of the zwiterion, also in solution [20].

Contrary to this coordination mode, HTBA coordinates to the gold(I) ion through its deprotonated form with the negative charge to be located at the sulphur atom forming neutral complexes [21].

Extended intermolecular hydrogen bonding interactions {N2[H96] ⋯ S1^ii^ = 3.2692(17), N1[H97] ⋯ O1^I^ = 2.790(2), O3[H98] ⋯ O1^iii^ = 2.630(2), and O3[H99] ⋯ S1^iv^ = 3.2302(17) Å, respectively, where the symmetry transformations used to generate equivalent atoms are (i) −*x*+1, −*y*, −*z*+2, (ii) −*x*+2, −*y*, −*z*+2, (iii) *x*+1/2, −*y*+1/2, *z*−1/2, (iv) −*x*+3/2, *y*+1/2, −*z*+3/2} lead to the supramolecular assembly of the complex (Figure 1(b)).

The influence of complex **1** on the oxidation of linoleic acid by the enzyme **LOX** was studied in a wide concentration range. The degree of LOX activity (**A**, %) in the presence of the complex was calculated according to the method described previously [3–8]. Figure 2 gives the inhibitory effect of complex **1** at various concentrations. It is shown that the catalytic activity of **LOX** was decreased in the presence of low concentrations (about 5–75 *μ*M) of the complex **1** (IC_50_ = 25 *μ*M) while no such activity was shown for cisplatin [3–8]. These values are comparable to the ones found for other similar Sn(IV) complexes. For example, the IC_50_ values found for the organotin compounds tested towards LOX, were 26 and 14 *μ*M for [(C_6_H_5_)_2_SnCl(HMNA)] and [(C_6_H_5_)_3_Sn(MNA)Sn(C_6_H_5_)_3_ (acetone)] (H_2_MNA = 2-thiobarbituric acid), respectively [6], 19, 16, and 21 *μ*M for ([(C_6_H_5_)_3_Sn(MBZT)]), ([(C_6_H_5_)_3_Sn(MBZO)]), and ([(C_6_H_5_)_3_Sn(CMBZT)]) (MBZT = 2-mercapto-benzothiazole, MBZO = 2-mercapto-benzoxazole and CMBZT = 5-chloro-2-mercapto-benzothiazole), respectively [3], 10, 13, and 14 *μ*M for ([(C_6_H_5_)_2_Sn(CMBZT)_2_]), ([(*n*-C_4_H_9_)_2_Sn(CMBZT)_2_]), and ([(CH_3_)_2_Sn(CMBZT)_2_]), respectively [3] and 61.3, 26.2, 20.5, and 16.9 *μ*M for ([(CH_3_)_2_Sn(PMT)_2_]), ([(*n*-C_4_H_9_)_2_Sn(PMT)_2_]), ([(C_6_H_5_)_2_Sn(PMT)_2_]), and ([(C_6_H_5_)_3_Sn(PMT)]) (PMT = 2mercapto-pyrimidine), respectively [4].

In order to investigate further the complex-protein interactions, we performed computational molecular docking studies for the complexes. The binding energy (*E*) of the substrate (S = linoleic acid) to its binding site in the enzyme LOX (**E**) when **ES** is the complex formed was *E* = −7.89 kcal/mole [6]. The corresponding binding energies of the inhibitor (**I**) are calculated to −6.92 and −6.37 kcal/mol for **ESI** and **EI,** respectively. Figure 3 shows the binding site of compound **1** towards LOX. Compound **1** binds to both **ESI** and **EI** complexes at the same pocket where the strong inhibitors of LOX bind [3], supporting its strong inhibition activity, found experimentally. Since high inhibition activity of LOX has been detected for all cytotoxic organotin(IV)-thione compounds tested previously, [3–8] such a strong activity is also expected for this compound.

Complex **1** was also tested for antitumor potential against sarcoma cancer cells (mesenchymal tissue) from the Wistar rat, polycyclic aromatic hydrocarbons (PAH, benzo[a]pyrene) carcinogenesis. Cytotoxic activity for complex **1** was evaluated as % percentage of the cell survived in variable concentrations 50 to 1000 nM (or 0.05 to 1 *μ*M) of the complex after 24 hours. The IC_50_ value found for **1** was 125 nM or 0.125 *μ*M indicating very strong cytotoxic activity against leiomyosarcoma cells as compared to cisplatin (IC_50_ = 4-5 *μ*M [8]). The corresponding IC_50_ values of other organotin(IV) complexes found against leiomyosarcoma cells were 0.005 *μ*M for [(C_6_H_5_)_3_Sn(MNA)Sn(C_6_H_5_)_3_ (acetone)] [7], 1.5–3, 1.3–3, and 0.5–0.8 *μ*M for ([(C_6_H_5_)_3_Sn(MBZT)]), ([(C_6_H_5_)_3_Sn(MBZO)]), and ([(C_6_H_5_)_3_Sn(CMBZT)]) (MBZT = 2-mercapto-benzothiazole, MBZO = 2-mercapto-benzoxazole and CMBZT = 5-chloro-2-mercapto-benzothiazole), respectively [3], 0.3–0.5, 0.6–0.8, and 5–7.5 *μ*M for ([(C_6_H_5_)_2_Sn(CMBZT)_2_]), ([(*n*-C_4_H_9_)_2_Sn(CMBZT)_2_]), and ([(CH_3_)_2_Sn(CMBZT)_2_]), respectively [3] and 20–60, 0.7, 1-2, and 0.1 *μ*M for ([(CH_3_)_2_Sn(PMT)_2_]), ([(*n*-C_4_H_9_)_2_Sn(PMT)_2_]), ([(C_6_H_5_)_2_Sn(PMT)_2_]), and ([(C_6_H_5_)_3_Sn(PMT)]) (PMT = 2mercapto-pyrimidine), respectively [4].

## Figures and Tables

**Scheme 1:**
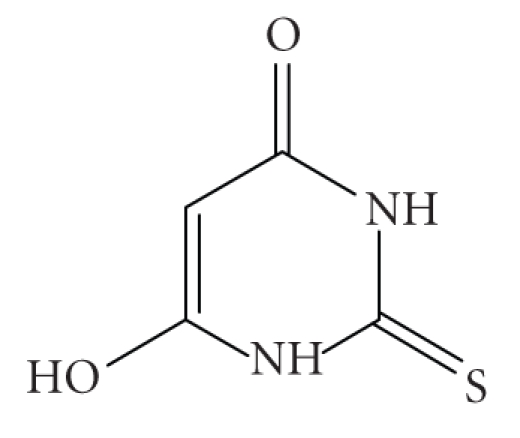


**Figure 1:**
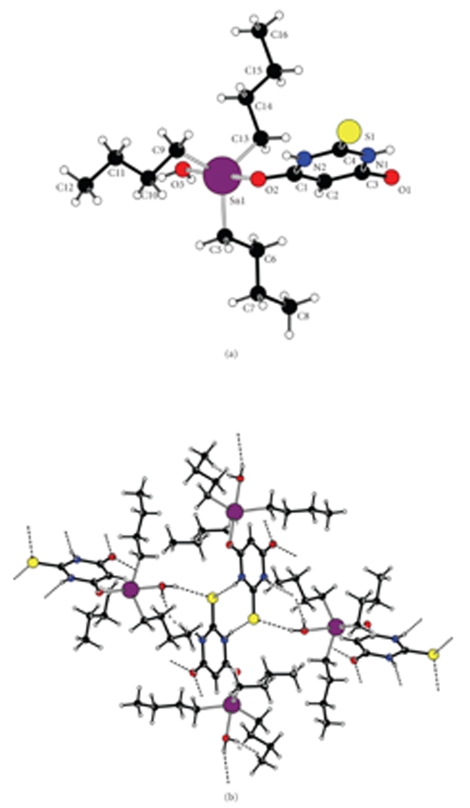
(a) Diagram of compound **1** together with the atomic numbering scheme. Selected bond lengths (Å) and angles [°]; Sn1–O2 = 2.2287(14), Sn1–O1 = 2.3410(15), Sn1–C5 = 2.136(2), Sn1–C9 = 2.1372(18), Sn1–C13 = 2.1408(19), C1–O2 = 1.274(2), C1–C2 = 1.386(3), C2–C3 = 1.394(3), C3–O1 = 1.261(2), C1–N2 = 1.389(3), C3–N1 = 1.393(3), C5–Sn1–C9 = 120.41(8), C5–Sn1–C13 = 120.97(8), C9–Sn1–C13 = 118.24(8), C5–Sn1–O2 = 91.12(7), C9–Sn1–O2 = 89.76(7), C13–Sn1–O2 = 95.23(7), C5–Sn1–O3 = 91.14(7), C9–Sn1–O3 = 87.87(7), C13–Sn1–O3 = 84.81(7), O2–Sn1–O3 = 177.33(6). (b) 3D hydrogen bonded network.

**Scheme 2:**
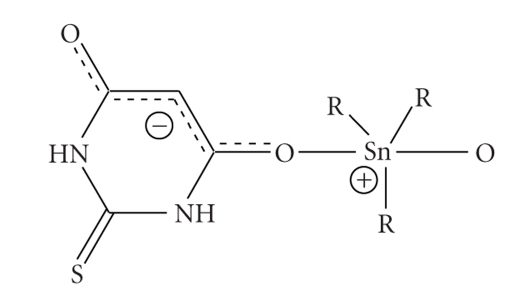
A possible bond and charge distribution on the atoms in **1**.

**Figure 2:**
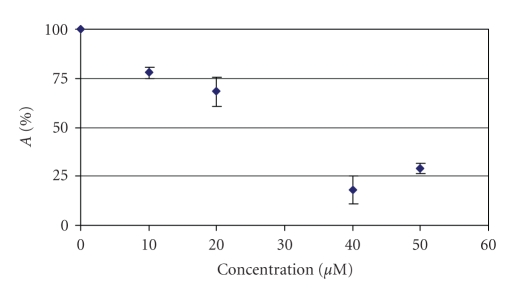
Inhibitory effect of **1** towards **LOX**.

**Figure 3:**
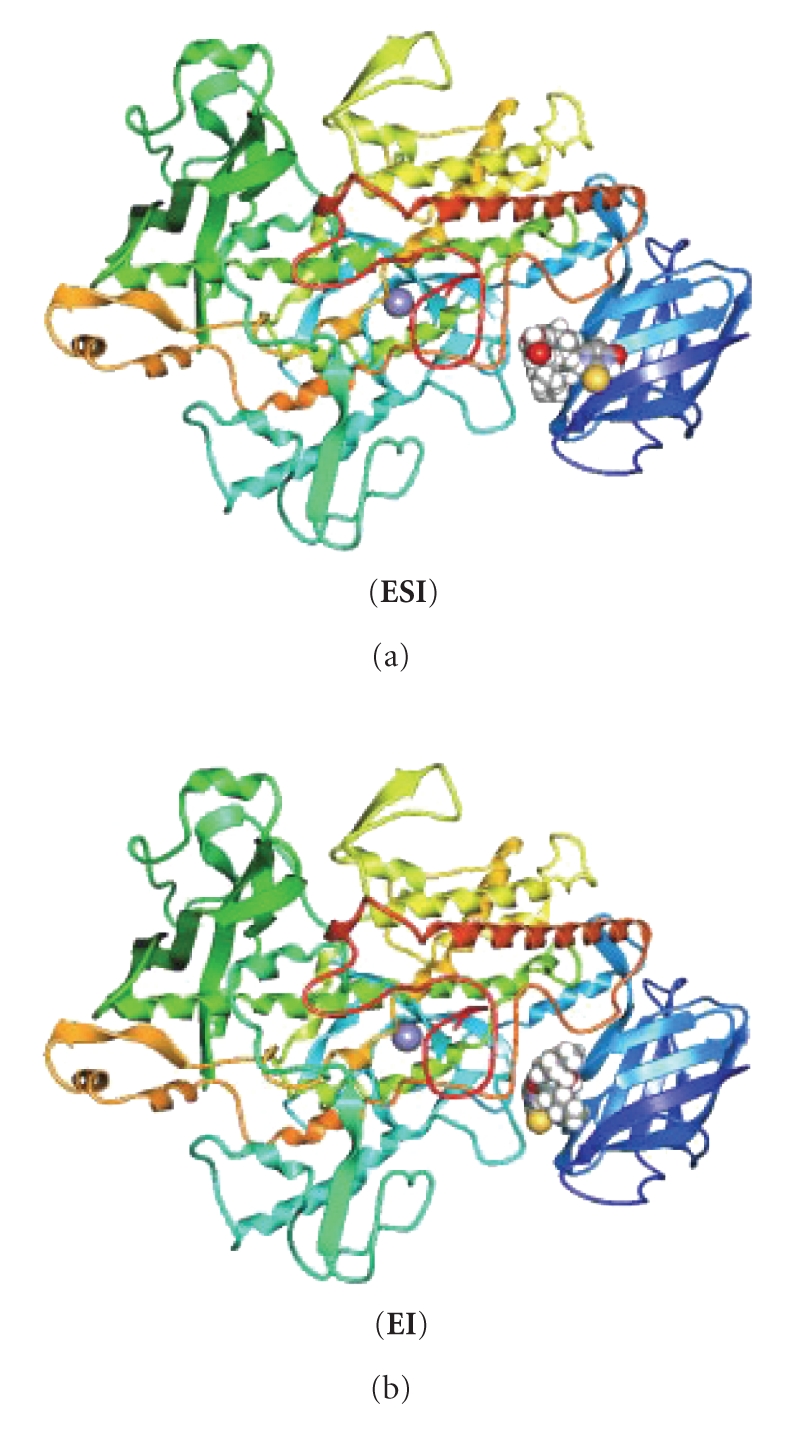
Binding sites of inhibitor **1** in lipoxygenase. The “walls” of this binding pocket concist of the amino acid residues: **ESI** : 76ALA, 533ARG, 767ARG, 128ASN, 769ASN, 760ASP, 768ASP, 78GLU, 75GLY, 249LEU, 110LYS, 15MET, 108PHE, 762VAL; **EI** : 76ALA, 533ARG, 767ARG, 128ASN, 760ASP, 768ASP, 761GLU, 247GLY, 248HIS, 246LEU, 249LEU, 110LYS, 762VAL.
